
JOURNAL INFORMATION
==============================
NLM Title Abbreviation: Atlanta Med Surg J (1884)
NLM Journal ID: 101251623

Atlanta Medical and Surgical Journal (1884)

Publisher: Biomedical Journal Digitization Project

ARTICLE INFORMATION
==============================
PMCID: PMC9201867
PMID: 35829613
Article version: 1
Subjects: Original Communications

Abortion; Its Prevention and Treatment* Paper read before the Atlanta Society of Medicine, January 19, 1899.

Smith W. Monroe M.D. Atlanta, Ga.

Print publication date: 1899 Feb
Volume: 15
Volume Series: New Ser
Issue: 12
First page: 803
Last page: 807
License: This work is free of known copyright restrictions. For more information, please see PMC Back Issue Digitization.
License URL: https://creativecommons.org/publicdomain/mark/1.0/

==============================

not often that a positive diagnosis becomes necessary in the early months, and by the time later symptoms establish the diagnosis beyond a doubt, the woman will already have made a diagnosis for herself. It is, therefore, my rule during the first three months of pregnancy, when consulted by a patient for the purpose of determining this point, to reserve my opinion, to avoid committing myself, and to wait for time to render a verdict from which there can be no appeal.

---

### ABORTION; ITS PREVENTION AND TREATMENT.*

By W. MONROE SMITH, M.D.,

ATLANTA, GA.

By abortion is generally meant the expulsion of the ovum before the formation of the placenta, which is complete at the end of the third month. But, in this paper, I desire to include by the term abortion the expulsion of the fetus during the first three or four months of gestation.

The study of the method of conception, development of the ovum and its membranes, the reception of the ovum by the uterus, the formation of the decidua and nourishment of the ovum, is of the most intense interest, but does not belong to the domain of this paper, and will not be discussed.

Abortion in all of its phases is a condition which every general practitioner must treat, and there is nothing which the physician encounters that is dreaded more by the young doctor than is abortion.

From personal experience abortion is very frequent—*i. e.*, relatively speaking. Abortion is thought by some writers to occur some time in the life of almost every married woman. Some women abort frequently. It occurs most frequently in women who have borne several children, but is by no means rare in primiparæ.

Predisposing causes of abortion may be either fetal, maternal or paternal.

---

*Paper read before the Atlanta Society of Medicine, January 19, 1899.

Maternal causes may be endometritis, retrodisplacement of the uterus, laceration of cervix with inflammation, excessive coitus, coughing, falls, blows, diseases of the decidua, syphilis, febrile affections from the eruptive diseases or from typhoid or malarial poisons, etc.

The paternal causes are syphilis and excessive coitus.

Fetal causes are diseases of the appendages (amnion, chorion, cord-placenta). The most frequent fetal cause of abortion is perhaps syphilitic degeneration of the villi, which cuts off the nourishment of the ovum, causing its death and expulsion. When once the attachment of the ovum to the uterus has been rendered insecure from any of these numerous causes, accidents which, in the absence of the predisposing condition would be insignificant, suffice to terminate the pregnancy.

Changes in the ovum, other than rupture and escape of the amniotic fluid, rarely lead at once and directly to abortion.

The exciting causes which induce uterine contractions and expulsion of the ovum reside, for the most part, in the maternal system.

When the predisposing causes have operated to weaken the attachment of the ovum to the decidua, anything which determines the blood current to the uterus is liable to produce extravasations of blood around the ovum and thus awaken uterine contractions.

Because of this fact, patients predisposed to above should be surrounded with every precaution during the periodic menstrual congestion, which not even pregnancy altogether suspends.

Fevers from any cause, inflammatory condition of the genital organs, excessive coitus, valvular heart disease, obstructions to the circulation of the lungs and liver, hot foot-baths, may each lead to rupture of the decidual vessels.

Most frequently, however, rupture follows jars to the body from coughing, falls, railroad journeys, from vomiting, violent exercise and the like.

Symptoms: As the detachment and expulsion of the ovum cannot possibly take place without rupture of the decidual and placental vessels, hemorrhage becomes the constant and necessary result and symptom of every abortion. In the first few weeks the symptoms resemble an obstrusive and profuse menstruation. After the second month there are usually prodromal symptoms, as fullness and weight in the pelvis, sacral pains, frequent micturition, periodic labor pains, with some watery discharge. These symptoms, with history of cessation of the menses and some hemorrhage, suggest a threatened abortion. If the hemorrhage be slight, it may cease and the pregnancy go on undisturbed. But oftener the hemorrhage increases in amount and uterine contractions set in and the ovum is expelled.

Usually during the first few weeks of pregnancy the ovum and the membranes are expelled entire.

Where abnormal adhesions attach the vera and serotina to the uterus, retained portions of the membranes may remain after the ovum has been expelled.

In another class, and this is the rule after the third month, the fetal membranes rupture, and the fetus escapes with the liquor amnii. The retained portions may soon follow the expulsion of the fetus. But it frequently happens that the uterus becomes inert or retracts, the os closes and period of repose follows. This constitutes an incomplete abortion.

Most frequently hemorrhage continues until the retained membranes are expelled, but sometimes the hemorrhage ceases for days, or weeks, and then reappears. If the membranes remain any length of time, putrid decomposition is quite likely to set in and cause septic infection, and sometimes produces death.

The diagnosis is based upon pain, hemorrhage, dilatation of the cervix and decent of the ovum. When the ovum can be felt through the os, the diagnosis is certain. It is hardly probable that a polypus would be mistaken for an ovum.

Prognosis should usually be good, if the case is properly treated. If the case is neglected or improperly treated, the hemorrhage may continue and the patient die from acute anemia, or septic infection may set in and close the scene.

Preventive treatment consists in carefully considering the cause that predisposes to abortion and, so far as practicable, in removing it.

Women who frequently abort should keep the bed during each menstrual period. If there be retroflexion or retroversion of the uterus, it should be replaced, and a properly fitting pessary introduced.

Lacerated and inflamed cervix should be properly treated.

Where abortion has occurred several times, and in the absence of other reasonable cause, syphilis may be suspected, and the iodides, with mercury, should be administered.

If abortion threatens, patient must be kept in bed, uterine and nervous sedatives freely given. In my hands, morphine has given me the best results as a nervous sedative. Viburnun is highly recommended by some writers as a specific in threatened abortion, but it often causes nausea and vomiting, for which reason I have abandoned its use.

I have used aletris quite often as a uterine sedative and tonic in this condition, and have been uniformly pleased with the results. It seldom, if ever, causes nausea and vomiting. But regardless of the most careful and skillful treatment, abortion will often occur.

When abortion becomes inevitable, as evidenced by pain, continued and free hemorrhage, dilatation of the cervix and decent of the ovum, the treatment should be that which will promptly empty the uterus. If the uterus does not promptly empty itself, especially if hemorrhage is considerable, the cervix should be dilated, and the ovum and membranes removed with the finger or dull curette.

C loroform is given if necessary, or ether if preferred. If, for any reason, the uterus cannot be emptied at once, and hemorrhage is threatening, the vagina should be thoroughly packed with absorbent cotton made into tampons, first soaked in two per cent. solution of carbolic acid and squeezed dry. Tampons should be tied together with cord, to facilitate removal. The tampons may be left in place ten or twelve hours if necessary; but, where choice can be had, it is much preferable to at once empty the uterus, when all anxiety for patient's safety should be over.

In incomplete abortion the uterus should be thoroughly curetted with dull curette, and, if hemorrhage is troublesome, should be packed with iodoform gauze for twelve or twenty-four hours. In all cases where the uterus is emptied artificially it should be done under the most strict asepsis and thoroughly drenched with bichloride or other good antiseptic hot solution.

The after-treatment consists in rest in bed and attention to such details as contribute to the comfort of the patient. Ergot may be given to aid retraction of uterus and assist involution.

---
